# TIFA suppresses hepatocellular carcinoma progression via MALT1-dependent and -independent signaling pathways

**DOI:** 10.1038/sigtrans.2016.13

**Published:** 2016-07-22

**Authors:** Wenzhi Shen, Renle Du, Jun Li, Xiaohe Luo, Shuangtao Zhao, Antao Chang, Wei Zhou, Ruifang Gao, Dehong Luo, Juan Wang, Na Hao, Yanhua Liu, Yanan Chen, Yunping Luo, Peiqing Sun, Shengyong Yang, Na Luo, Rong Xiang

**Affiliations:** 1 Department of Immunology, School of Medicine, Nankai University, Tianjin, China; 2 International Joint Center for Biomedical Research of the Ministry of Education, Tianjin, China; 3 Department of Immunology, Institute of Basic Medical Science, Chinese Academy of Medical Science and Peking Union Medical College, Beijing, China; 4 Department of Cancer Biology and Comprehensive Cancer Center, Wake Forest University Medical Center, Winston-Salem, North Carolina, USA; 5 West China Hospital, Molecular Medicine Research Centre, State Key Lab Biotherapy, Sichuan University, Chengdu, China

## Abstract

TIFA, also called T2BP, was first identified using yeast two-hybrid screening. Our previous work showed that TIFA suppresses hepatocellular carcinoma (HCC) progression via apoptosis and cell cycle arrest. However, the mechanism by which this TIFA suppression occurs remains unclear. Here we demonstrated that TIFA-induced apoptosis demonstrates two distinct time patterns (i.e., at 48 h and >7 days) when TIFA reconstitution occurs. Moreover, we found that MALT1 (a competitor of TIFA) plays a crucial role in short-duration TIFA reconstitution. In this regard, MALT1 silencing with shRNA markedly enhances TIFA-induced apoptosis *in vitro* and *in vivo*. In addition, TIFA overexpression triggers JNK and p38 activation in long-duration TIFA reconstitution through TRAF6 binding. In particular, JNK activation leads to TIFA-induced apoptosis while p38 activation governs TIFA-induced cell cycle arrest by p53-p21 signaling *in vitro* and *in vivo*. Our data suggest a novel mechanism by which TIFA suppresses HCC progression via both MALT1-dependent and MALT1-independent signaling pathways. This may provide insights into a novel targets where HCC progression may be vulnerable to clinical treatment.

## Introduction

Chronic liver inflammation is associated with an increased incidence of liver cancer.^1^ The most common type of liver cancer is hepatocellular carcinoma (HCC), which is an end product of chronic liver disease or inflammation and typically requires decades to evolve. Various inflammatory mediators are known to participate in the development of HCC. Therefore, an analysis of inflammatory signaling pathways may reveal markers or targets to identify or treat patients with chronic liver inflammation, particularly those predictive of HCC.

TIFA (TNF receptor associated factor (TRAF)-interacting protein with a Forkhead-associated (FHA) domain), also called T2BP,^2^ was first identified as a novel protein that interacts with TRAF6 using yeast two-hybrid screening.^3^ TIFA contains a FHA domain (which directly binds phosphothreonine and phosphoserine) and a consensus TRAF6-binding motif. TIFA overexpression promotes oligomerization and poly-ubiquitinylation of TRAF6, which in turn activates TAK1 and IKK.^4,5^ The TAK1 and IKK activation links TRAF6 to NF-κB in the IL-1/TLR4 pathway.^6^ Recent studies also demonstrated that TIFA oligomerization was indispensable for innate immunity induced by bacterial metabolite HBP (heptose-1,7-bisphosphate).^7,8^ Our previous findings showed that: (a) TIFA expression is suppressed during HCC progression and (b) TIFA reconstitution induced the expression of p53 thereby promoting apoptosis, while suppressing proliferation among surviving cells. These previous findings implicate TIFA as a previously unappreciated suppressor of liver carcinogenesis via p53-dependent/independent mechanisms and provide insight into a vulnerability of HCC.^1^

Malt1 is a multi-domain cytosolic signaling molecule that was originally identified as the target of recurrent translocations in a large fraction of MALT lymphomas.^9^ The human paracaspase MALT1 is a caspase homolog that has a central role in NF-κB signaling.^10^ The action of MALT1 is owing to a combination of its scaffolding and proteolytic function.^11^ In addition, MALT1 targets key proteins in a feedback loop that mediates termination of the NF-κB response and thus promotes activation of NF-κB signaling.^10^ The caspase-like domain of Malt1 (amino acids 326–567) binds to caspase-8 with Leu 359 essential for the biochemical association between Malt1 and caspase-8.^12^ Furthermore, the C-terminal region of Malt1 contains two binding sites for TRAF6 and TRAF2 that center around Glu653 and Glu806,^13^ although only the most C-terminal TRAF binding site may be functional.^14,15^ In addition, the binding of MALT1 with caspase-8 and TRAF6 contribute to the activation of the NF-κB signaling pathway.^9^ However, the function of MALT1 in HCC remains unexplored.

In this study, we investigated the role of MALT1 in TIFA-induced apoptosis in HCC cell lines. We found that TIFA competes with MALT1 for TRAF6 binding and that TIFA reconstitution induces MALT1 downregulation by 48 h then MALT1 levels return to normal by >7 days. In addition, we found that suppression of MALT1 via shRNA-silencing promotes TIFA-induced apoptosis, which depends upon caspase-8 activation. Furthermore, TIFA expression activated JNK and p38 signaling at >7 days. The activated JNK promotes apoptosis via JNK-caspase-8 signaling and activated p38 promotes cell cycle arrest via p53-p21 signaling. These findings suggest that TIFA suppresses HCC progression via with or without MALT1 ways.

## Materials and methods

### Vector construction

ShRNA targeting human MALT1 and scrambled control sequence were designed and chemically synthesized as MALT1-shRNA (5′-AAAAGCCTGTGTCTGCTGAAGTTAATTGGATCCAATTAACTTCAGCAGACACAGGC-3′). The control, TIFA-sc was 5′-AAAAGCTACACTATCGAGCAATTTTGGATCCAAAATTGCTCGATAGTGTAGC-3′. The palindromic DNA oligos were annealed to each other to form a double-strand oligo and ligated to the linearized pLV-H1-EF1α-puro (cat. #SORT-B19, Biosettia Inc.) vector to generate circled pLV-H1-shRNA-MALT1-Puro.

### Cell culture

Human HCC cell line, wt of SK-Hep1 cell line was kindly provided by Dr Ralph A Reisfeld (The Scripps Research Institute, La Jolla, CA, USA). WT of HepG2 was obtained from the Chinese Academy of Sciences. SK-Hep1-Wt and HepG2-Wt cells were infected with lentivirus carrying pLV-EF1α-Flag-TIFA-IRES-Bsd and pLV-EF1α-Flag-TIFAΔ6-IRES-Bsd plasmids, followed by clonal selection using Blasticidin (5 μg ml^−1^ for SKHep1 and HepG2) to generate polyclonal cell populations with stable overexpression of Flag-TIFA and TIFAΔ6 (Sk-Hep1-Flag-TIFA, Sk-Hep1-Flag- TIFAΔ6 and HepG2-Flag-TIFA, HepG2-Flag-TIFAΔ6). Alternatively, SK-Hep1-TIFA cells were infected with lentivirus carrying pLV-H1-MALT1-puro or scrambled shRNA plasmid, followed by clonal selection using 1 μg ml^−1^ puromycin to generate polyclonal cell populations with stable expression of shMALT1 or sc. SK-Hep1 and HepG2 cells were maintained in Dulbecco's modified Eagle's medium supplemented with 10% fetal bovine serum, 100 U ml^−1^ penicillin/streptomycin and 1% NEAA.

### Immunoblotting

Cell lysates from different cell lines were prepared with RIPA buffer in the presence of protease inhibitor cocktails and Phosphatase Inhibitor Cocktail 2 and 3 (P8340, P5726 and P0044, Sigma-Aldrich, St Louis, MO, USA). Protein (30 μg) was loaded onto 8–15% Tris-acrylamide gels and blotted with antibodies that included: anti-TIFA (Cat. #ab124956), p-JNK (cat. #ab32142), p38 (cat. #ab103011 Abcam Biotechnology, Inc., Abcam, Hong Kong, China); MALT1, (cat. # 04–580 Millipore, Billerica, MA, USA); β-actin (cat. #sc-47778), p53 (cat. #sc-126), JNK (cat. #sc-571, Santa Cruz Biotechnology, Inc. Santa Cruz, CA, USA); caspase 3 (cat. #9665), TAK1 (cat. #4505), p-TAK1 (Thr184/187) (cat. #4508), caspase-8 (cat. #9496s, Cell Signal Technology Inc., Danvers, MA, USA); ERK (cat. # ZS-94), p-ERK (cat. # ZS-7976), p-p38 (cat. # ZS-101759, ZSGB-BIO) and horseradish peroxidase-conjugated secondary antibodies. Blotting results were detected by an ECL chemiluminescence kit (cat. #17153, Millipore, Billerica, MA, USA).

### Pathscan intracellular signaling array

The cell lysates were prepared with 1× Cell Lysis Buffer from the kit, dilute lysates to 1.0 mg ml^−1^ in Array Diluent Buffer. Here we loaded 70 μg protein for this experiment, all assay procedures were performed according to the manufacturer’s directions (cat. #7323, Cell Signal Technology Inc.).

### Flow cytometry analysis of cell cycle and apoptosis

Following culture of cells in the absence of fetal bovine serum for 12 h, cells were ‘pulsed’ with 10 mm 5-bromo-2-deoxyuridine for 24 h, and cell cycle assay was performed by using the Cytofix/cytoperm kit (BD Biosciences, San Jose, CA, USA) following the manufacturer’s instructions. For apoptosis assay, apoptotic cells were stained with propidium iodide and Annexin V-FITC (BD Biosciences). Flow cytometry analysis was performed by FACS Calibur cytometer (BD Biosciences), in which a minimum of 10 000 events were recorded. Three independent assays were conducted in such experiments and the mean values were expressed as mean ±s.e.m.

### Tumor xenografts

Male NOD/SCID mice at 6–8 weeks of age were separated randomly into three groups (*n*=5) for each group based on minimal 30% decrease from 1 g tumors with 250 μg s.d., *α* error of 0.05 and a *β* error of 0.8). In all, 3×10^6^ SK-Hep1 cells (SK-Hep1-Flag-TIFA-sc, SK-Hep1-Flag-TIFA-shMALT1) were inoculated subcutaneously into each mouse at right axilla. Tumor volume (mm^3^) was measured with calipers two times per week and calculated by using the standard formula: length×width^2^/2. The individual measuring the mice was unaware of the identity of the group measured. Animal use complied with Nankai University Animal Welfare Guidelines.

### Inhibitors treatment

SK-Hep1-TIFA cells were seeded at a density of 0.5×10^6^ per 60 mm dish. Twenty-four hours after seeding, the cells were treated with 10 μm PD98059 (ERKi), 1 μm JNK420119 (JNKi), 10 μm SB203580 (p38i) and dimethyl sulfoxide used as the Ctrl for 48 h, then cells were harvested for FACS or protein extraction. For the *in vivo* tests, male NOD/SCID mice at 6–8 weeks of age were separated randomly into three groups (*n*=5), 3×10^6^ SK-Hep1-TIFA cells were inoculated subcutaneously into each mouse at right axilla. After 10 days, mice were intraperitoneally injected JNK420119 (40 mg kg^−1^), SB203580 (20 mg kg^−1^) and dimethyl sulfoxide injected as the control.

### Immunohistochemistry

Immunostaining was performed on paraffin human HCC tissue samples. Expression levels of TIFA or MALT1 in the tissue samples were scored according to the percentage of TIFA or MALT1-positive cells in each liver tissues. The images were recorded by Olympus BX51 Epi-fluorescent microscopy under a ×10 or ×40 objective (Olympus Co., Tokyo, Japan). All tissues have signed the informed consent with patients before using.

### TUNEL staining

Paraffin-embedded tissue slides were prepared from the tumor xenografts DeadEnd Fluorometric TUNEL System kit (Promega, Madison, WI, USA) was applied for TUNEL staining. Experiment procedure was performed according to the manuscript instruction. 4,6-Diamidino-2-phenylindole was used to stain the nuclei, and the tissue slides were subjected to Olympus BX51 Epi-fluorescent microscopy under a ×40 objective (FV1000-IX81, Olympus Microsystems, Shanghai, China).

### Statistical analysis

Values were expressed as means±s.e.m. Significance was determined by *χ* ^2^-test, others were determined by Student’s *t*-test. A value of *P*<0.05 was used as the criterion for statistical significance. * Indicates significant difference with *P*<0.05, ** Indicates significant difference with *P*<0.01, *** Indicates significant difference with *P*<0.001.

## Results

### TIFA expression is decreased in HCC

**Figure 1:**
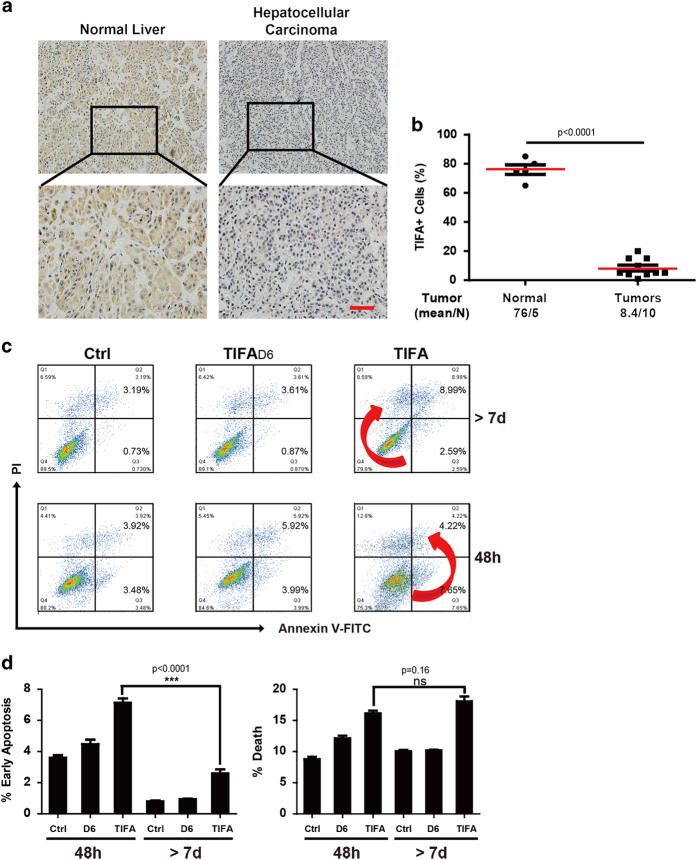
Validation of TIFA function in HCC. (**a**) The representative image of TIFA staining in human tissues (including 10 HCC samples and 5 normal samples). Scar bar, 100 μm. (**b**) The relationship of TIFA-positive cells with tissue status. Each point represents one sample and the middle line represents the mean value. Data are shown as the mean±s.e.m. The number of samples and the mean values of each group are listed below the *x* axis. (*P*-value was determined by Student’s *t*-test). (**c**) Flow cytometry showing the percentage of dead (*Q*_D_=*Q*_1_+*Q*_2_) or early apoptosis (*Q*_A_=*Q*_3_) cells detected by PI-Annexin V double staining in TIFA or TIFAΔ6 cells. Cells were collected after infected with virus for 48 h or >7d. A representative experiment of three is shown. TIFAΔ6 means TRAF6 binding site mutation which does not bind TRAF6. (**d**) Statistical results of the percentage of dead or apoptotic cells are shown. d, days; HCC, hepatocellular carcinoma.

In order to confirm our previous finding that TIFA expression is decreased in HCC, we examined TIFA expression levels in liver biopsies from 15 patients (10 HCC and 5 normal biopsies) using immunocytochemistry. We found that TIFA- immunostaining is qualitatively decreased in HCC biopsies versus normal liver biopsies (Figure 1a). In addition, a quantitative analysis of the TIFA- immunostaining shows that the percent of TIFA-immunopositive cells is decreased in the HCC biopsies versus normal liver biopsies (8.4% vs 76.0%, respectively; Figure 1b). The above-mentioned results confirm our previous finding that TIFA is indeed decreased in HCC.

### TIFA reconstitution fosters distinct apoptotic patterns in a time-dependent manner

In order to further confirm the functional role of TIFA in HCC, we examined apoptosis in the HCC tumor cell line SK-Hep-1 following TIFA reconstitution by lentivirus for 48 h and after several passages post infection (>7 days) using (PI-Annexin V double staining) flow cytometry. Non-reconstituted SK-Hep1 cells (Ctrl) and TIFA∆6-reconstituted SK-Hep1 cells served as controls. The results indicate that TIFA-reconstituted SK-Hep1 cells at 48 h post-reconstitution undergo changes from normal (*Q*_4_) to early apoptosis (*Q*_3_=7.65%), then to late apoptosis, and finally to cell death (*Q*_D_=*Q*_1_+*Q*_2_=12.8%+4.22%=17.02%; Figure 1c, red arrow). However, TIFA-reconstituted SK-Hep1 cells at >7 days post-reconstitution interestedly undergo changes from normal to cell death directly (*Q*_D_=*Q*_1_+*Q*_2_=8.59+8.99%=17.58%; Figure 1c, red arrow) without seemingly passing through early apoptosis (*Q*_3_). The above-mentioned differences at 48 h and >7 days post-reconstitution are supported by our observation that the percentage of TIFA-reconstituted SK-Hep1 cells at 48 h post-reconstitution in early apoptosis is higher than the percentage TIFA-reconstituted SK-Hep1 cells at >7 days post-reconstitution in early apoptosis (*Q*_3_; 7.65% vs 2.59%, respectively; Figure 1d). However, the percentage of TIFA-reconstituted SK-Hep1 cells at 48 h post-reconstitution in cell death is the same as the percentage of TIFA-reconstituted SK-Hep1 cells at >7 days post-reconstitution in cell death (*Q*_D_=*Q*_1_+*Q*_2_; 17.02% vs 17.58%, respectively; Figure 1d). The observation that TIFA reconstitution fosters distinct apoptotic patterns in a time-dependent manner may open a new avenue of study concerning the mechanism of TIFA function and apoptosis.

### MALT1 mediates the inhibition of TIFA-induced apoptosis *in vitro*

**Figure 2:**
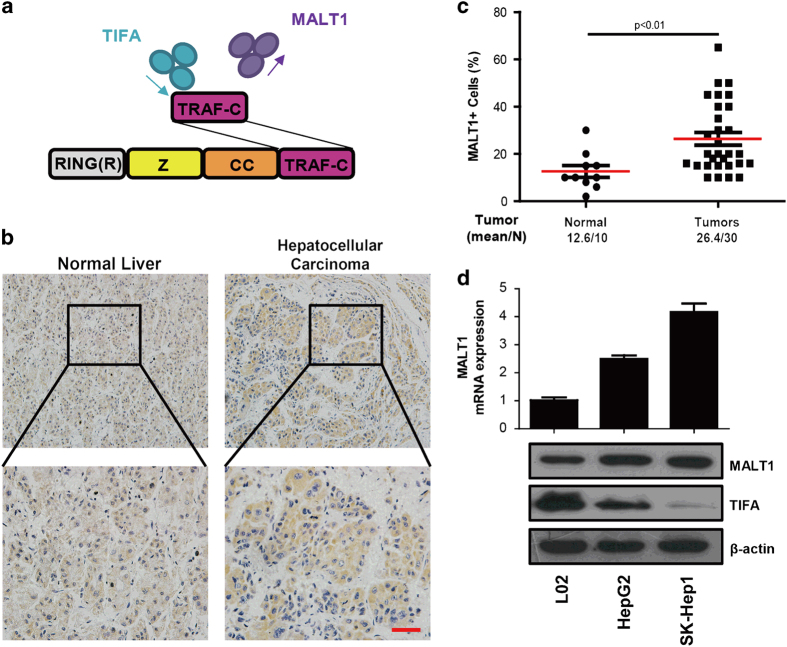
MALT1 was increased in HCC along with TIFA decreased. (**a**) Model of TIFA and MALT1 Binding to TRAF6. (**b**) The representative image of MALT1 staining in human tissues (including 10 HCC samples and 5 normal samples). Scar bar, 100 μm. (**c**) The relationship of MALT1-positive cells with tissue status. Each point represents one sample and the middle line represents the mean value. Data are shown as the mean ±s.e.m. The number of samples and the mean values of each group are listed below the *x* axis. (*P*-value was determined by Student’s *t*-test). (**d**) qPCR and western blot were performed to analysis MALT1 expression in RNA and protein levels. HCC, hepatocellular carcinoma; qPCR, quantitative PCR.

In order to investigate the time-dependent TIFA-induced apoptosis, we sought to identify TIFA-related proteins that could structurally or functionally effect TIFA signaling. MALT1 manifested as a strong candidate since MALT1 competitively binds with TIFA for the TRAF-C domain of TRAF6 (ref. 16) and may undergo self-oligomerization (Figure 2a). We examined MALT1 expression levels in liver biopsies from 15 patients (10 HCC and 5 normal biopsies) using immunocytochemistry. We found that MALT1-immunostaining is qualitatively increased in HCC biopsies versus normal liver biopsies (Figure 2b). In addition, a quantitative analysis of the MALT1-immunostaining shows that the percent of MALT1-positive cells is increased in HCC biopsies versus normal liver biopsies (15.1% vs 8.2%, respectively; Figure 2c). We also found that MALT1 expression is upregulated in HepG2 and SK-Hep1 liver cancer cell lines versus LO2 normal liver cell line both in RNA and protein levels contrary to TIFA (Figure 2d). These results indicate that TIFA and MALT1 expression levels show a reverse pattern; that is, when TIFA expression is high MALT1 expression is low and vice versa.

**Figure 3:**
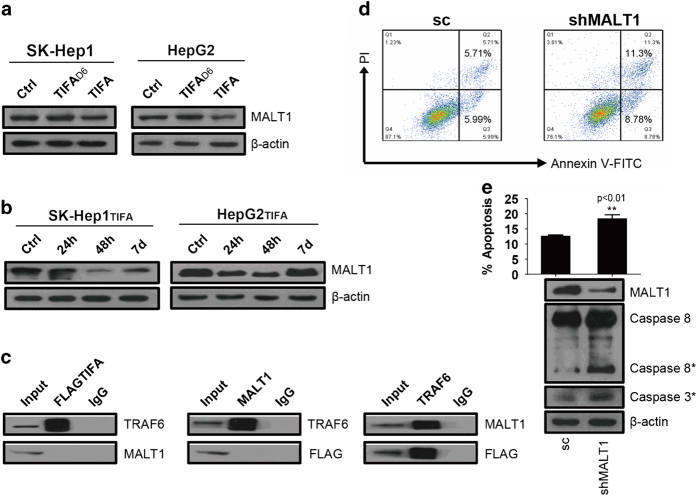
MALT1 inhibits apoptosis through competitively binding to TRAF6 with TIFA *in vitro*. (**a**) Western blot was performed to analysis MALT1 expression after ectopic expression of TIFA. (**b**) Western blot analysis MALT1 expression in different time points after virus infection. (**c**) Immunoprecipitation shows the competitive relationship of TIFA and MALT1. (**d**) Flow cytometry showing the percentage of apoptosis cells detected by PI-Annexin V double staining in SK-Hep1-TIFA-Ctrl or SK-Hep1-TIFA-shMALT1 cells. A representative experiment of three is shown. (**e**) Statistical results of the percentage of apoptotic cells are shown. The immunoblotting shows relative MALT1 expression as well as the expression of cleaved caspase-8 and caspase 3. β-actin is included as a loading control.

On the basis of the above-mentioned evidence, we then analyzed MALT1 expression after TIFA reconstitution in SK-Hep1 and HepG2 cell lines after several passages using western blot analysis. We found that MALT1 is expressed after TIFA reconstitution in both the SK-Hep1 and HepG2 cell lines (Figure 3a).

Next, we studied MALT1 expression after TIFA reconstitution in SK-Hep1-TIFA and HepG2-TIFA cell lines at 24 h, 48 h and 7 days post infection versus controls using western blot analysis. The results indicate that MALT1 expression decreases at 48 h and then returns to control levels at 7 days (Figure 3b). This suggests that ectopically expressed TIFA competitively binds to TRAF6 with MALT1 and thereby decreases MALT1 expression initially followed by a later return of MALT1 expression to control levels. This may play a role in TIFA-induced apoptosis. In order to further confirm the relationship of TIFA, MALT1 and TRAF6, we performed an immunoprecipitation assay using a SK-Hep1-TIFA cell line. As expected, we found that FLAG-TIFA antibody and MALT1 antibody pulled down only TRAF6. Whereas TRAF6 antibody pulled down both FLAG-TIFA and MALT1 (Figure 3c).

In order to elucidate more details about the mechanism by which MALT1 influences TIFA-induced apoptosis, we suppressed MALT1 expression in a SK-Hep1-TIFA cell line using short-hairpin RNA-mediated silencing (shMALT1). The results indicate that both early and late phase apoptosis increase (that is, cells do not proceed directly to cell death) in shMALT1-treated cells versus controls (Figure 3d). In addition, we observed the cleavage of procaspase-8 and increased levels of executioner caspase 3 in shMALT1-treated cells versus controls (Figure 3e). Taken together, these results suggest that MALT1 mediates the inhibition of TIFA-induced apoptosis along specific pathways and that this mediation may be caspase-8 dependent.

### MALT1 mediates the inhibition of TIFA-induced apoptosis *in vivo*

**Figure 4:**
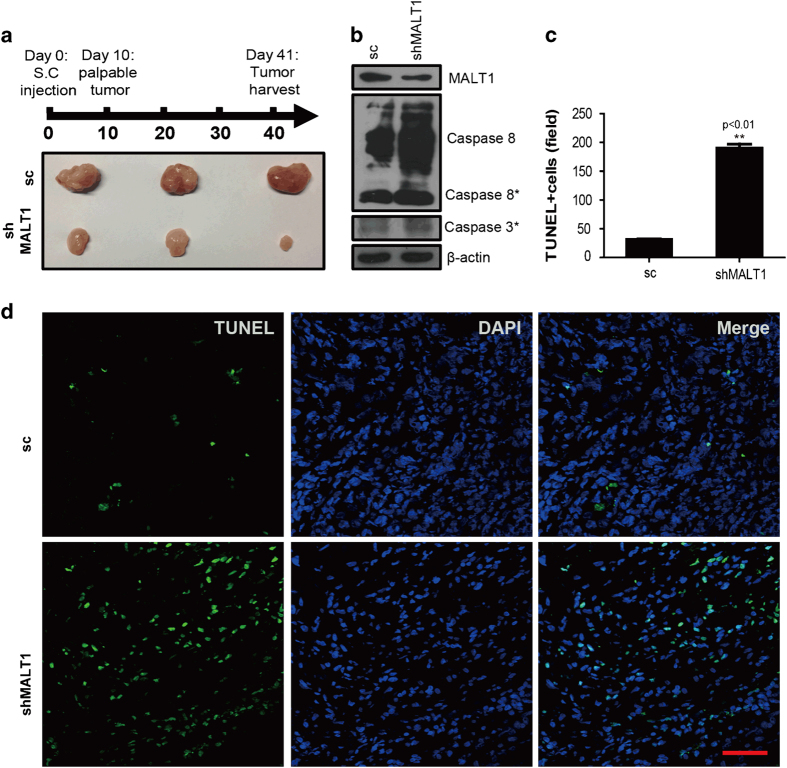
Silencing MALT1 enhances TIFA-induced apoptosis *in vivo*. (**a**) Up panel is the schema of establishing HCC mouse model via the subcutaneous implantation of SK-Hep1 cells. Down panel showing several tumors separated from two groups are shown. (**b**) Representative images showing TUNEL staining of xenografted tumor tissue from two groups (control, shMALT1). (**c**) Bar graph shows the statistical results of TUNEL staining illustrated in **c**. Scar bar, 100 μm. (**d**) Immunoblot analysis of the relative MALT1 expression as well as the expression of cleaved caspase-8 and caspase 3.

In order to determine whether MALT1 also inhibits TIFA-induced apoptosis *in vivo*, we performed tumor xenograft studies using SK-Hep1-TIFA-shMALT1 cells and SK-Hep1-TIFA-sc cells transplanted into NOD/SCID mice. The results indicate that the tumor size at Day 41 was markedly diminished in the SK-Hep1-TIFA-shMALT1 tumors versus the SK-Hep1-TIFA-sc tumors (Figure 4a). Concordant with the diminished tumor size, we observed that deoxynucleotidyl transferase dUTP nick end labeling (TUNEL) staining which detects DNA fragmentation consistent with apoptosis is both qualitatively (Figure 4b) and quantitatively increased in the SK-Hep1-TIFA-shMALT1 tumors versus the SK-Hep1-TIFA-sc tumors (190 TUNEL-positive cells vs 31 TUNEL-positive cells, respectively; Figure 4c). Finally, we observed the cleavage of procaspase-8 and increased levels of executioner caspase 3 in SK-Hep1-TIFA-shMALT1 tumors versus the SK-Hep1-TIFA-sc tumors (Figure 4d). The above-mentioned findings strongly suggest that MALT1 mediates the inhibition of TIFA-induced apoptosis *in vivo* and that this mediation may be caspase-8 dependent. These *in vivo* observations are consistent with our *in vitro* observations.

### JNK mediates TIFA-induced apoptosis through a caspase-dependent pathway

**Figure 5:**
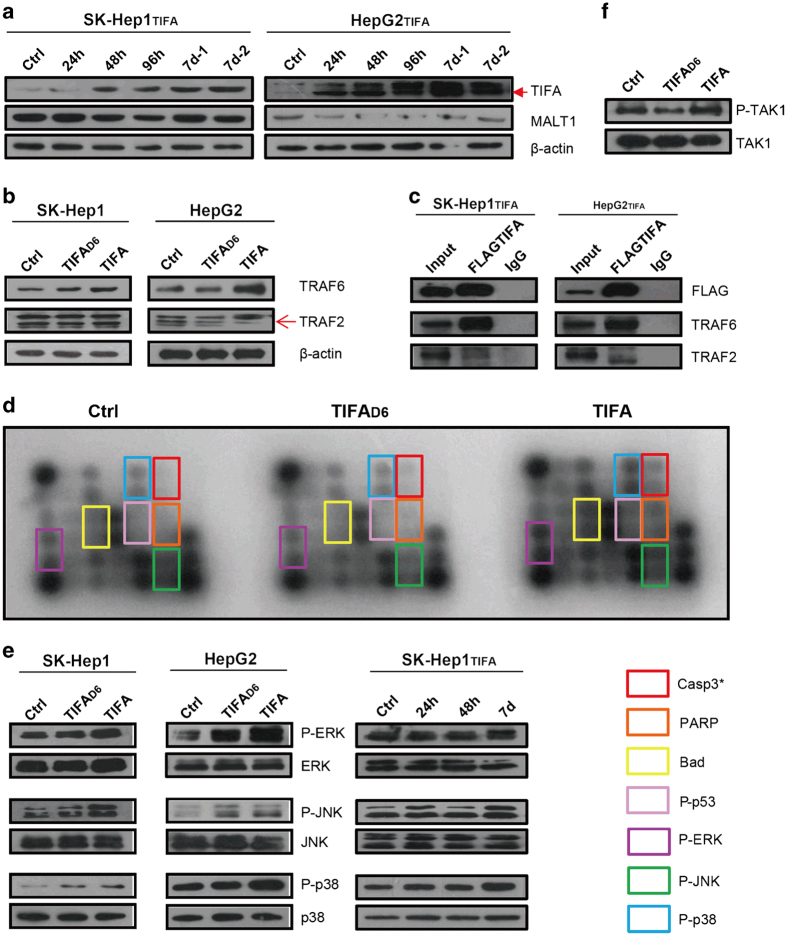
Ectopic expression of TIFA activates JNK, and p38 via TRAF6 binding. (**a**) The expression of TIFA and MALT1 was studied in different time points by immunoblot. (**b**) Western blot analysis of TRAF6 and TRAF2 expression after ectopic expression of TIFA. (**c**) Immunoprecipitation shows the TIFA binds to TRAF6 preferentially. (**d**) Pathscan array were performed to analysis the variation of key moleculars in different signaling pathways by TIFA induced. (**e**) Western blots were used to confirm ERK, JNK, p38 activation from pathscan results. (**f**) Western blots were used to confirm the TAK1 signaling activation.

In order to further investigate what happened after MALT1 expression returns to control levels at 7 days, first the TIFA and MALT1 expression was ensured in more time points (cells infected with TIFA virus at 0 h, 24 h, 48 h, 96 h and 7 days without puromycin selection-1 and 7 days with puromycin selection-2; Figure 5a), and found that MALT1 expression returns to control levels at 7 days exactly. Then we used the proteins after TIFA virus infected 7 days to investigated the levels of TRAF2 and TRAF6 (that is, binding proteins of TIFA) in SK-Hep1-TIFA cells and in HepG2-TIFA cells using western blot analysis. Non-reconstituted SK-Hep1 and HepG2 cells (Ctrl) and TIFA∆6-reconstituted SK-Hep1 and HepG2 cells served as controls. The results indicate that TRAF2 levels remain unchanged but TRAF6 levels increase (Figure 5b). In order to further confirm the relationship of TRAF6 and TIFA, we studied the binding of TRAF6 and TIFA in SK-Hep1-TIFA and HepG2-TIFA cell lines using immunoprecipitation. We found that FLAG-TIFA antibody pulled down TRAF6 but not TRAF2 (Figure 5c).

Next, we surveyed the levels of various apoptotic signaling proteins in SK-Hep1-TIFA cells versus non-reconstituted SK-Hep1 cells (Ctrl) and TIFA∆6-reconstituted SK-Hep1 cells using a pathscan array, which contained 19 diverse signaling proteins. The results show that the expression levels of not only cleaved caspase 3, cleaved PARP, and Bad (that is, apoptosis-associated proteins) increase, but also the expression levels of p-p53, p-ERK, p-JNK and p-p38 increase in SK-Hep1-TIFA cells versus controls (Figure 5d). The pathscan arrays results for p-ERK, p-JNK and p-p38 were confirmed in SK-Hep1 and HepG2 cell lines using western blot analysis (Figure 4e, left side). In addition, we studied the expression levels of p-ERK, p-JNK and p-p38 at 24 h, 48 h and >7 days post-reconstitution in SK-Hep1-TIFA cells using western blot analysis. We found that the expression levels of p-ERK, p-JNK and p-p38 were elevated at >7 days versus 24 and 48 h (Figure 5e, right side).

**Figure 6:**
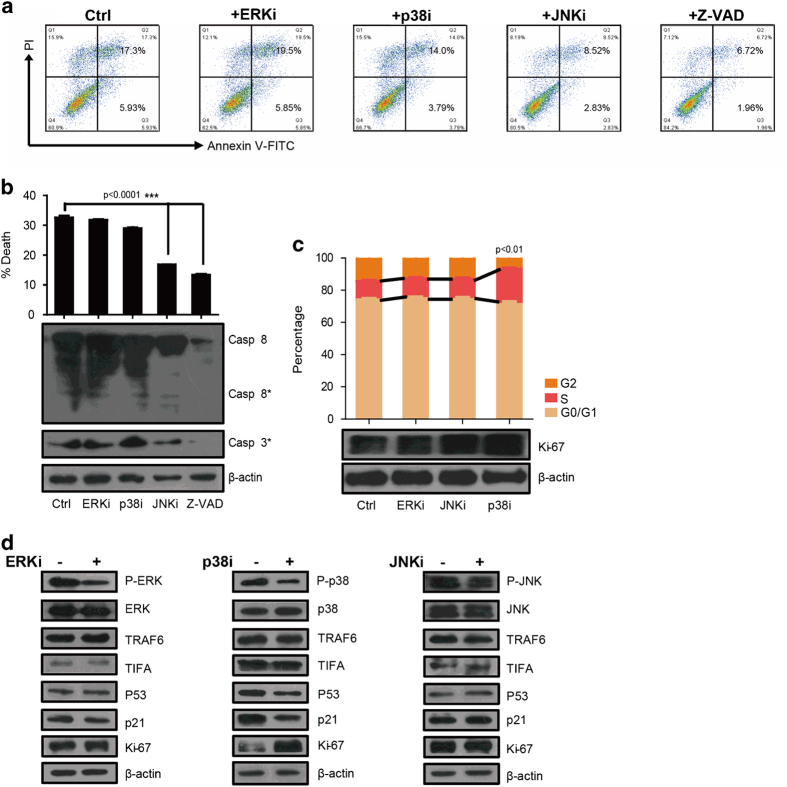
P-JNK mediates TIFA-induced apoptosis and p-p38 mediates TIFA-induced cell cycle arrest *in vitro*. (**a**) Flow cytometry shows the percentage of apoptosis cells detected by PI-Annexin V double staining in SK-Hep1-TIFA cells treated with ERK inhibitor, JNK inhibitor, p38 inhibitor and Z-VAD. A representative experiment of three is shown. (**b**) Statistical results of the percentage of apoptotic cells are shown. The immunoblotting shows relative expression of cleaved caspase-8 and caspase 3. β-actin is included as a loading control. (**c**) The bar graph of up panel shows the result of three separated experiments of cell cycle analysis via 7-aminoactinomycin D (7-AAD)/5-bromo-2-deoxyuridine (BrdU)-FITC double staining analyzed by flow cytometry. The bottom panel show the relative Ki-67 expression by immunoblot. (**d**) Western blot analysis of downstream signaling associated with s-phase increase treated with different inhibitors (PD98059: ERK inhibitor, SB203580: p38 inhibitor and JNK420119: JNK inhibitor).

The signaling proteins ERK, JNK, and p38 are all activated by the ectopic expression of TIFA through TAK1 activation (Figure 5f). However, which or what combination of these signaling proteins (that is, ERK, JNK or p38) have a direct role in TIFA-induced apoptosis remains unclear. In order to address this issue, we inhibited the activation of ERK, JNK and p38 (using ERKi, JNKi and p38i) for 48 h in SK-Hep1-TIFA cells and observed apoptosis using (PI-Annexin V double staining) flow cytometry. Untreated SK-Hep1-TIFA cells and Z-VAD (a caspase inhibitor)-treated SK-Hep1-TIFA cells served as controls. We found that JNK inhibition markedly decreased the level of apoptosis in SK-Hep1-TIFA cells (Figure 6a). Whereas ERK inhibition and P38 inhibition showed no difference in the level of apoptosis compared with untreated SK-Hep1-TIFA cells (controls). The above-mentioned results were confirmed by a decrease in the percentage of dead cells and a corresponding decrease in caspase-8 and caspase 3 activation in the JNKi-treated SK-Hep1-TIFA cells (Figure 6b). These results suggest that JNK activation has a specific role in TIFA-induced apoptosis via a caspase-8 dependent pathway.

### P38 mediates TIFA-induced cell cycle arrest through p53-p21 signaling

In order to investigate the mechanism involved in TIFA-induced cell cycle arrest, we inhibited the activation of ERK, JNK and p38 (using ERKi, JNKi and p38i) for 48 h in SK-Hep1-TIFA cells and observed the cell cycle phases using 7-aminoactinomycin D (7-AAD)/5-bromo-2-deoxyuridine (BrdU)-FITC double staining analyzed by flow cytometry. The results demonstrate that p38 inhibition increases the S phase population of cells (Figure 6c). Moreover, the increase in the S phase population of cells was accompanied by an increase in Ki-67 and a decrease in both p53 and p21 (Figure 6d). The above results suggest that p38 activation mediates TIFA-induced cell cycle arrest through a p53-p21 signaling pathway.

### JNK and p38 mediate similar effects *in vivo* as found *in vitro*

**Figure 7:**
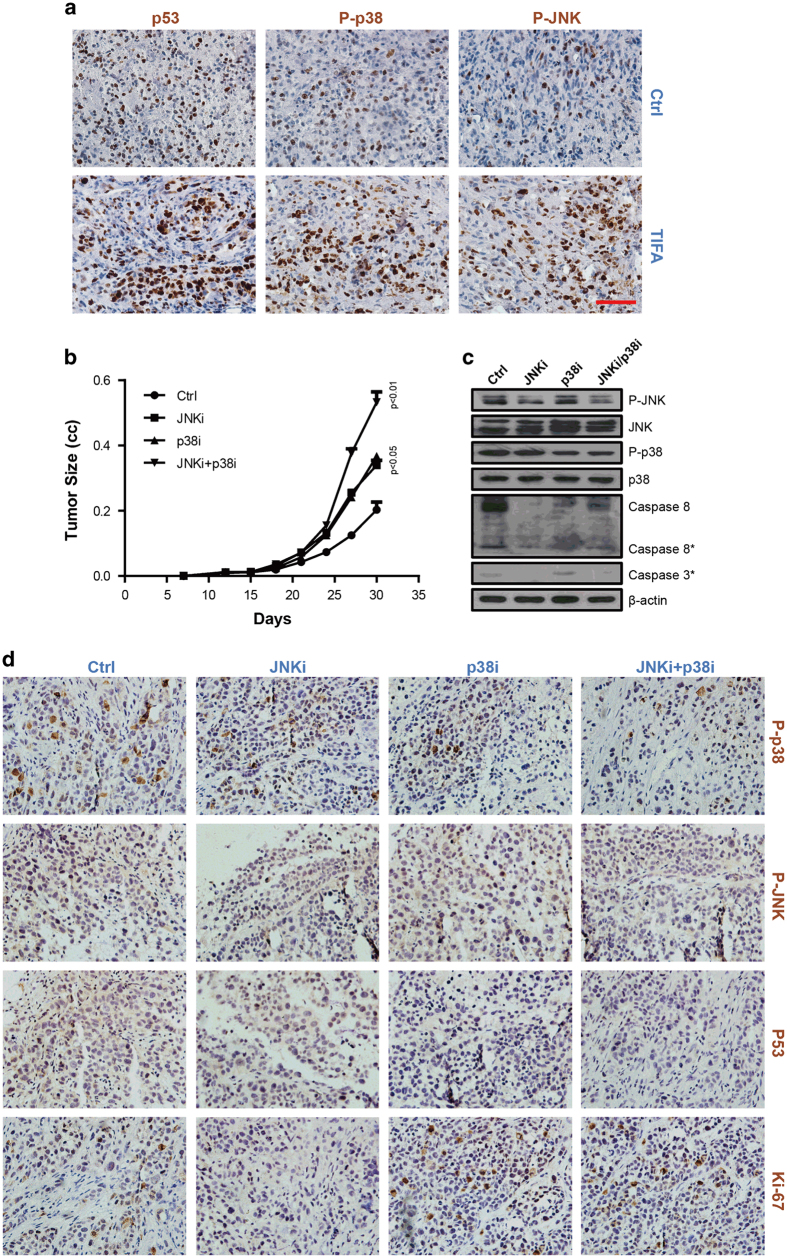
P-JNK mediates TIFA-induced apoptosis and p-p38 mediates TIFA-induced cell cycle arrest *in vivo*. (**a**) The representative photomicrographs show that p-JNK-, p-p38-, and p53-immunostaining is qualitatively increased in SK-Hep1-TIFA tumors versus SK-Hep1 tumors (Ctrl). Scale bar, 100 μm. (**b**) Tumor growth curve of SK-Hep1-TIFA cells treated with control, JNKi, p38i and JNKi/p38i tumors harvested from the flank of NOD/SCID mice. (**c**) Immunoblot analysis of the expression of p-JNK, p-p38, caspase-8 and cleaved caspase 3 in SK-Hep1-TIFA tumors treated with control, JNKi, p38i and JNKi/p38i, β-actin is included as a loading control. (**d**) The images show the immunohistochemistry staining of p-JNK, p-p38, p53 and Ki-67 in tumor tissues separated from xenografted NOD/SCID mice subcutaneously injected with SK-Hep1-TIFA cells treated with control, JNKi, p38i and JNKi/p38i.

In order to validate our *in vitro* results concerning JNK and p38, we localized p-JNK, p-p38 and p53 in tumors from xenografted NOD/SCID mice subcutaneously injected with SK-Hep1-TIFA cells or SK-Hep1 cells (control) using immunocytochemistry. We found that p-JNK, p-p38 and p53-immunostaining qualitatively increased in the SK-Hep1-TFIA tumors versus the SK-Hep1 (control) tumors which is consistent with our *in vitro* observations (Figure 7a). At the same time, to determine the effects of inhibitors were maintained *in vivo*, NOD/SCID mice subcutaneously injected with SK-Hep1-TIFA cells treated with dimethyl sulfoxide (control), JNKi and p38i were performed to analyze the tumor size, cell proliferation and cell apoptosis. Consistent with the results *in vivo*, a significantly large tumor size was observed among tumors treated with JNKi and p38i (Figure 7b). Concordant with the enlarged tumors, we observed increased ki-67 level as a result of lower levels of p-p38 and p53 (Figure 7c). Moreover, decreased levels of cleaved caspase-8 and caspase 3 were found in JNKi groups (Figure 7d). These *in vivo* findings lend support to the notion that JNK and p38 inhibit TIFA-induced tumor progression.

### TIFA induces distinct apoptotic pathways through TIFA-TRAF6-MALT1 interaction

**Figure 8:**
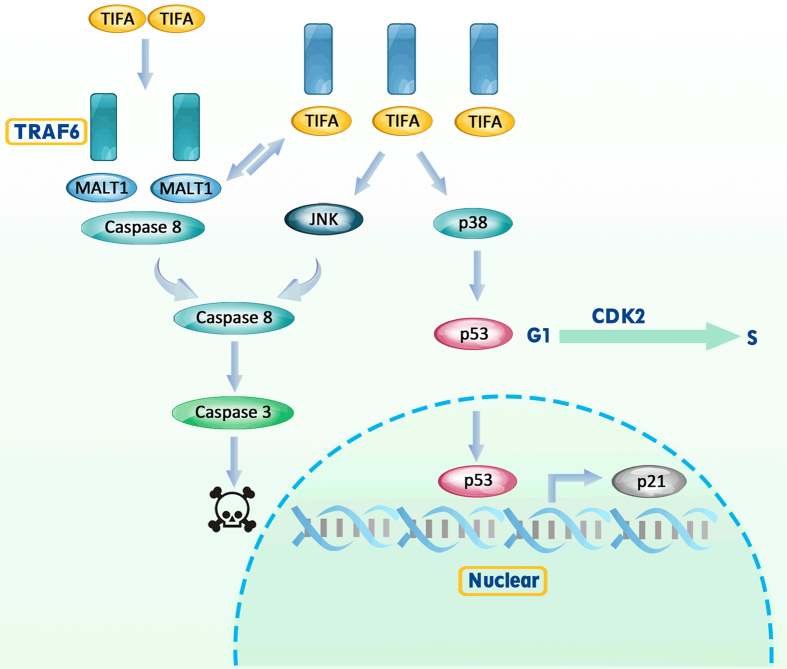
A proposed model of TIFA- and MALT1-Mediated apoptosis and cell cycle arrest.

Our findings can be summarized in a flow chart that indicates the TIFA-TRAF6-MALT1 interactions and their resultant effects (Figure 8). The process starts as MALT1 undergoes self-oligomerization and competitively binds to TRAF6 (specifically the TRAF-C domain) along with TIFA. MALT1 works as an adapter between TRAF6 and caspase-8 which blocks the cleavage of caspase-8. With the ectopic expression of TIFA at 48 h, TIFA competes with MALT1 for TRAF6 binding which frees caspase-8 for easy cleavage leading to apoptosis. With the ectopic expression of TIFA at >7 days, TIFA and MALT1 establish a balanced binding with TRAF6. As TIFA continues to increase, more TRAF6 is synthesized which acts as an adapter to start downstream signaling. The downstream signaling activates JNK and p38. JNK activation causes caspase-8 cleavage and leads to cell apoptosis. P38 activation recruits p53 and p21 signaling and leads to cell cycle arrest.

## Discussion

HCC is the third most common cause for cancer-related death worldwide.^17^ HCC has become the most prevalent cause for cancer-related deaths in some African and Asian countries.^18^ In addition, clinical success in the pharmacological treatment of HCC patients has been limited. Currently, early-stage diagnosis of HCC may be the best way to improve the prognosis of HCC. Unfortunately, most HCCs are usually diagnosed at later stages with the main therapeutic methods consisting of surgery and chemotherapy. Therefore, the identification of new targets or predictive markers for HCC therapy remains urgent.

Our findings concerning the changes in MALT1 expression levels and their effect on TIFA-induced apoptosis were unexpected. Previous studies from other labs revealed that MALT1 has a central role in NF-κB signaling via proteolytic cleavage of A20 and the proteolytic cleavage of caspase-8 to c-FLIP_L_, which enhances NF-κB signaling.^19,20^ Many previous studies have shown that MALT1 acts as an oncogene that promotes the progression of cancers (for example, diffuse large B-cell lymphomas and lung cancer).^21–24^ Here we present evidence that MALT1 promotes the progression of HCC by the inhibition of TIFA-induced apoptosis. We suggest that MALT1 competitively binds to TRAF6 (specifically the TRAF-C domain) along with TIFA. With TIFA overexpression at 48 h, TIFA competes with MALT1 for TRAF6 binding, which frees caspase-8 for easy cleavage opening an avenue to apoptosis. However, with TIFA overexpression at >7 days, a feedback signaling pathway (for example, the NF-κB signaling pathway) maybe triggered that upregulates MALT1 to original levels. This opens an alternate avenue to apoptosis via JNK and caspase-8. The proposition that two distinct avenues to apoptosis involving caspase-8 exist may be the unresolved issue in this study. In this regard, MALT1 downregulation may activate some other signaling pathway besides the NF-κB signaling pathway which ultimately results in caspase-8 activation. This warrants further investigation.

Our previous work has shown that TIFA-induced apoptosis depends on caspase activation (that is, not p53 accumulation), although the mechanism remains unresolved.^1^ TIFA expression also promotes activation of mitogen-activated protein kinase, extracellular signal-regulated kinase, c-JUN N-terminal kinase and p38. In particular, the activation of c-JUN N-terminal kinase and p38 triggers downstream cascades that lead to inflammation, cell differentiation or cell death.^25–29^ We used mitogen-activated protein kinase inhibitors to reveal two independent signaling pathways both of which were induced by TIFA. Interestingly, JNK activation led to TIFA-induced apoptosis whereas p38 activation governed TIFA-induced cell cycle arrest via p53-p21 signaling.

This study revealed two unexpected findings. Firstly, MALT1 which promotes and is regulated by NF-κB signaling is involved in TIFA-induced apoptosis by competitively binding to TRAF6 (specifically the TRAF-C domain) at 48 h. Silencing MALT1 enhances TIFA-induced apoptosis via caspase-8 activation. Secondly, TIFA reconstitution activates JNK and p38. JNK activation leads to TIFA-induced apoptosis whereas p38 activation governs TIFA-induced cell cycle arrest via p53-p21 signaling. Consequently, we have revealed a novel mechanism by which TIFA suppresses HCC progression via both MALT1-dependent and MALT1-independent signaling pathways. This may provide insights into targets where HCC progression may be vulnerable to clinical treatment.
