# Correction to: TIFA suppresses hepatocellular carcinoma progression via MALT1-dependent and -independent signaling pathways

**DOI:** 10.1038/s41392-025-02187-x

**Published:** 2025-03-04

**Authors:** Wenzhi Shen, Renle Du, Jun Li, Xiaohe Luo, Shuangtao Zhao, Antao Chang, Wei Zhou, Ruifang Gao, Dehong Luo, Juan Wang, Na Hao, Yanhua Liu, Yanan Chen, Yunping Luo, Peiqing Sun, Shengyong Yang, Na Luo, Rong Xiang

**Affiliations:** 1 Department of Immunology, School of Medicine, Nankai University, Tianjin, China; 2 International Joint Center for Biomedical Research of the Ministry of Education, Tianjin, China; 3 Department of Immunology, Institute of Basic Medical Science, Chinese Academy of Medical Science and Peking Union Medical College, Beijing, China; 4 Department of Cancer Biology and Comprehensive Cancer Center, Wake Forest University Medical Center, Winston-Salem, NC USA; 5 West China Hospital, Molecular Medicine Research Centre, State Key Lab Biotherapy, Sichuan University, Chengdu, China

Correction to: *Signal Transduction and Targeted Therapy* 10.1038/sigtrans.2016.13, published online 22 July 2016

Subsequent to the online publication of the article,^1^ the authors identified an unintentional error in Figure 7d. Specifically, the p-p38 data for the JNKi group partially overlaps with the Ki-67 data for the JNKi+p38i group, and the Ki-67 data for the p38i group also partially overlaps with the Ki-67 data for the JNKi+p38i group. This oversight arose from our failure to accurately extract and process the experimental data, resulting in the erroneous inclusion of two micrographs pertaining to the p-p38 protein within the Ki-67 protein folder. The corrected data are presented below. It is important to note that these amendments do not alter the key findings of the article.

Incorrect Figure:
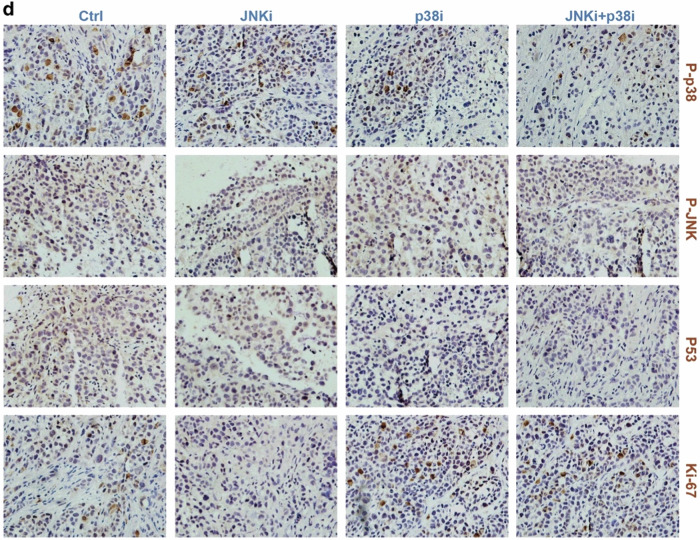


Correct Figure:
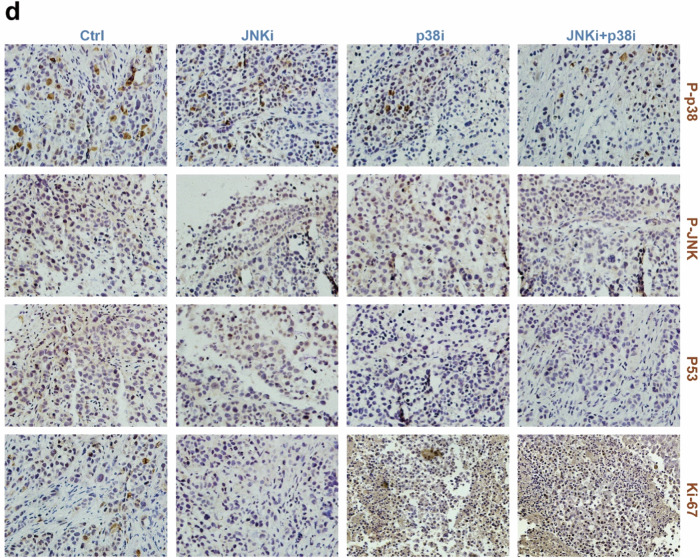


The original article has been corrected.
